# Folic Acid and Selected Risk Factors for Fetal Heart Defects—Preliminary Study Results

**DOI:** 10.3390/nu16173024

**Published:** 2024-09-06

**Authors:** Agnieszka Kolmaga, Elżbieta Trafalska, Ewelina Gaszyńska, Julia Murlewska, Sławomir Witkowski, Oskar Sylwestrzak, Łukasz Sokołowski, Maria Respondek-Liberska, Iwona Strzelecka

**Affiliations:** 1 Department of Nutrition and Epidemiology, Medical University of Lodz, 90-752 Lodz, Poland; 2 Department of Prenatal Cardiology, Polish Mother’s Memorial Hospital Research Institute in Lodz, 93-338 Lodz, Poland; 3 Department of Gynecology and Obstetrics, Polish Mother’s Memorial Hospital Research Institute in Lodz, 93-338 Lodz, Poland; 4 Department of Diagnoses and Prevention of Fetal Malformations, Medical University of Lodz, 90-419 Lodz, Poland

**Keywords:** pregnancy, congenital heart defects, folic acid, risk factors

## Abstract

Background: The available data on the relationship between diet/folic acid and congenital heart disease (CHD) are not consistent. This study aimed to investigate the relationship between the intake and supplementation of folic acid and other selected factors in mothers and the risk of congenital heart defects in fetuses. Methods: A case–control study was conducted. The study group included pregnant women with fetuses from singleton pregnancies with prenatally diagnosed heart defects in the fetus (*n* = 79) and pregnant women whose course of pregnancy was normal with no heart defects or other developmental anomalies diagnosed in the fetus (*n* = 121). The patients were diagnosed at a reference center in Poland. The women completed a lifestyle questionnaire and *FFQ* and precisely described their use of dietary supplements. A univariate logistic regression model was used to evaluate the association between folic acid and selected risk factors and CHD. The association was significant and included such risk factors such as nutritional status, medications taken, smoking, and alcohol consumption. Additionally, the time of starting folic acid supplementation turned out to be statistically significant. The reference period of supplementation was the period before pregnancy. Results: Lack of supplementation increases the risk of heart defects in children by more than four times compared to supplementation before pregnancy (OR = 4.19; *p* = 0.0117), whereas supplementation beyond the eighth week of gestation increases the risk almost threefold (OR = 2.90; *p* = 0.0474). The presence of congenital defects in the family is also an important factor. Conclusions: A history of congenital heart defects or other defects, lack of periconceptional folic acid supplementation, and lack of dietary supplementation before pregnancy were associated with congenital heart defects in the fetus. Place of residence, parents’ education, lifestyle habits such as smoking and alcohol consumption, nutritional status before pregnancy, and mother’s diseases did not show a significant relationship with congenital heart defects in the children. There is an urgent need to develop preventive strategies and conduct extensive public education.

## 1. Introduction

Proper nutrition for women before and during pregnancy is important for the course of pregnancy and optimal development of the fetus. A woman’s diet should provide all the necessary nutrients [1,2,3,4].

An extremely important period in the development of individual systems/organs (including the fetal heart) is the period of organogenesis. The development of this organ is a dynamic process that begins approximately 18–19 days after fertilization. The mechanisms of abnormal cardiac morphogenesis refer to disruption of the normal embryological process which occurs during the first 8 weeks of pregnancy [5,6,7,8]. At this time, congenital heart and vascular system defects may develop, which constitute the most common group of congenital malformations in Poland [9,10,11]. When it comes to prenatal patients, the incidence of heart defects in fetuses is 24:1000 pregnancies [12,13]. Defects in this population occur at least 10 times more often than the most common genetic defect syndromes (i.e., Down’s syndrome). It is important to remember that genetic defect syndromes most often involve cardiac issues.

Based on current data, it is believed that the majority of fetal heart defects may be caused by multiple environmental and genetic factors acting together [14,15,16]. The occurrence of genetic factors is estimated at 10–15%. The remaining factors involved in the development of heart defects in the fetus are related to environmental factors (including diet, alcohol consumption, cigarette smoking, medications, environmental pollution, etc.) [9]. Knowing and understanding the risk factors of congenital heart defects are very important for better understanding the etiology of this condition and establishing/developing preventive strategic actions. The identification of modifiable risk factors is primarily aimed at reducing the burden of the disease and ensuring health education for society by increasing knowledge and awareness, especially among women of reproductive age [17,18,19].

The scientific and research project entitled “The analysis of the relationship between a woman’s diet before and during pregnancy and the occurrence of congenital heart defects in the fetus” would allow for verification of the relationship between the consumption and supplementation of selected nutrients and the occurrence of fetal heart anomalies.

This study aimed to investigate the relationship between the intake and supplementation of folic acid and other selected factors in the mother and their association with congenital heart defects in the fetus. This research was conducted in the Department of Prenatal Cardiology of the Institute of the Polish Mother’s Health Center in Lodz and the Department of Diagnostics and Prevention of Congenital Defects of the Medical University of Lodz in cooperation with the employees from the Department of Nutrition and Epidemiology of the Medical University of Lodz. 

Based on data from clinical trials, significant risk factors associated with fetal heart defects were selected [12,14,15,16,17,20,21,22,23,24,25,26] and verified in this part of our own study. 

### 1.1. What Is New?

- To our knowledge, this is the first study of this type that verifies the relationship between the consumption and/or supplementation of folic acid and the occurrence of heart defects in fetuses in the population of pregnant women in Poland.
- The history of congenital heart defects or other defects, lack of periconceptional folic acid supplementation, and lack of dietary supplementation before pregnancy were associated with congenital heart defects in the newborns.
- The place of residence, parents’ education, lifestyle habits such as smoking, alcohol consumption, nutritional status before pregnancy, and mother’s diseases did not show a significant relationship with congenital heart defects in the newborns.

### 1.2. What Are the Clinical Implications?

- This study strengthens evidence regarding the significant relationship between the use of folic acid supplementation in the preconception period and supplementation of a diet low in folate as a protective factor against congenital heart defects in the fetus.
- There is an urgent need to develop preventive strategies and conduct broad public education aimed at women of reproductive age regarding folic acid supplementation as a complement to a diet low in folate in order to reduce the risk of congenital heart defects in the fetus.

### 1.3. Objective of the Work

The aim of the study was to assess and analyze the intake of folic acid from the diet and dietary supplementation and their association (including risk factors such as nutritional status, medications taken, smoking, and alcohol consumption) in individuals with a congenital heart defect in prenatal life, who made up the study group (A), and in pregnant women whose pregnancy showed no irregularities (normal heart anatomy and function), who made up control group (B). The occurrence of congenital defects in the family was also analyzed.

## 2. Materials and Methods

### 2.1. Study Group

The participants included pregnant women of the Department of Prenatal Cardiology of the Polish Mother’s Health Center in Łódź and the Department of Diagnostics and Prevention of Congenital Defects of the Medical University of Łódź from different places in Poland who were referred for detailed fetal heart diagnostics.

The study was conducted from February 2021 to January 2022 at a reference center for fetal defects in cooperation with the employees from the Department of Nutrition and Epidemiology of the Medical University of Lodz. A total of 660 pregnant women were examined, and 776 echocardiographic examinations were performed. Some 251 pregnant women were included in the analysis, and for the purposes of the current study, 200 patients were included.

The study group included pregnant women with their fetuses from singleton pregnancies with prenatally diagnosed heart defects in the fetus (*n* = 79) and pregnant women whose course of pregnancy was normal, with no heart defects or other developmental anomalies in the fetus diagnosed (*n* = 121). The women were in the second and third trimester of pregnancy and were aged 19–44.

### 2.2. Inclusion and Exclusion Criteria

The inclusion criteria for the study were pregnant women in good general health, without addictions, over 18 years of age. The exclusion criteria included multiple pregnancies and those from which unreliable data were obtained in nutritional interviews, any non-cardiac abnormalities detectable by ultrasound and echocardiography at an appropriate stage of pregnancy, and genetic defects in the fetus.

The research tool constituted a validated questionnaire used to assess the frequency of consumption of products and dishes during the last year before the study, with particular emphasis on the period before pregnancy (FFQ—Food Frequency Questionnaire). For the purposes of this study, this questionnaire was modified at the Department of Nutrition and Epidemiology of the Medical University of Lodz. Consumption data were verified by a 1-day interview on the consumption on the day before the examination (24 h recall). The questionnaires were completed independently by the pregnant woman on the day of the fetal echocardiologic examination according to the attached instructions and with the help of available tools (including a photo album of products and dishes) [27]. These data were verified with verification questions by a trained interviewer from the Department of Nutrition and Epidemiology during a direct conversation, or (due to the current epidemiological situation) by e-mail.

The interviews included the consumption of vitamin and mineral supplements used before and during pregnancy. The pregnant women were asked about the type, dose, and frequency of the supplement used, including single or combined preparations. The obtained results were prepared using the Dieta 6.0 computer program based on the database of the composition and nutritional value of food products developed by NIZP-PZH-PIB employees. The research methodology used, based on the above program, included so-called inevitable losses [28].

Then, the obtained data on the consumption of energy and nutrients—taking into account the age, gender, trimester of pregnancy and declared physical activity of the respondents—were compared with the current nutritional standards at the level of the group’s average requirement (EAR—Estimated Average Requirement) and the sufficient level of intake (AI—Adequate Intake) [1]. Risk factors such as nutritional status, medications taken, smoking, and alcohol consumption were also taken into consideration.

On the day of the fetal echocardiographic examination at the Department, the patients were asked to complete a questionnaire regarding their general health. The survey included questions regarding basic data, i.e., trimester of pregnancy, patient’s age, education, use of medications, accompanying diseases and defects occurring in the family.

The research material also included medical documentation and examination results collected at the Department of Prenatal Cardiology of the Institute of the Polish Mother’s Health Center in Łódź.

### 2.3. Statistical Analysis

Descriptive methods and statistical inference methods were used to analyze the data. Structure indices, arithmetic mean ($\bar{x}$), median (Me) as average measures, and standard deviation (SD) a measure of dispersion were calculated. The compliance of the distributions of the analyzed measurable variables with the normal distribution was based on the Shapiro–Wilk test. The non-parametric Mann–Whitney test was used to compare means, and the Pearson chi-square test was used to examine relationships between variables. Univariate logistic regression analysis was used to isolate factors that may be significantly associated with heart defects in the fetus. This regression model was used to analyze factors separately. To assess statistical significance, the error probability was *p* ≤ 0.05.

## 3. Results

There was no statistically significant difference in the age of the examined women in the analyzed groups (*p* > 0.005). The mean age in groups A and B was similar and was 31.9 ± 5.3 years and 31.4 ± 5.6 years, respectively.

However, a statistically significant difference in the age of the fetuses was found (*p* < 0.001). It turned out that in the NHA group, the mean age of the fetuses was significantly lower than in the CHD group: 26.7 ± 5.4 weeks vs. 29.6 ± 5.6 weeks (*Z* = 3.539; *p* = 0.0004). The data are presented in Table 1.

Comparison of groups A and B in terms of mean BMI values did not show a statistically significant difference; the means were similar and amounted to 24.7 ± 5.2 kg/m^2^ and 25.9 ± 6.3 kg/m^2^, respectively.

Groups A and B did not differ significantly in terms of mean values obtained with diet (*p* > 0.05). The average folate values obtained from the diet in both analyzed groups were below the recommended values [600 µg], although a slightly higher intake could be observed in the group of women with no fetal heart defects [428.1 µg ± 149.0], compared to the average intake in the group of women with fetal heart defects [392 µg ± 129.2]. The corresponding mean values from supplementation did not differ significantly between the groups (*p* > 0.05) and were as follows: in group A, they were 463.3 µg ± 178.0, and in group B, 492.4 µg ± 193.7. In the case of folate intake from diet and supplementation combined, the difference between the means in groups A and B was not statistically significant (*p* > 0.05). However, in group A, the mean was much lower than in group B (543.6 ± 365.2 vs. 614.5 ± 374.0). The data are presented in Table 2.

Only 14.36% of the surveyed pregnant women consumed sufficient folate from their diet. A higher percentage of pregnant women [19.83%] in whom fetal heart defects were not diagnosed met the folate intake requirements compared to pregnant women with diagnosed heart defects [15.19%]. A dietary intake of folic acid below the norm, compared to women whose intake was within the norm, was associated with the risk of heart defects in the child, although this was not statistically significant (OR = 1.48; *p* > 0.05). Detailed data are presented in Table 3.

Folic acid supplementation was taken by the women in the form of multivitamins (compounds) or pure folic acid preparations. Folic acid supplementation in the study group was declared by only 38.4% of pregnant women before pregnancy. Folic acid supplementation was used by 82.9% of pregnant women from group A, of which only 13.2% declared their use before pregnancy and 69.7% during pregnancy, while as many as 17.1% of the surveyed women in this group did not use folic acid supplementation. In the control group, 92.2% of women used dietary supplements with folic acid (including 25.2% before pregnancy and 67.0% during pregnancy). No supplementation was declared by 7.8% of women in group B.

The time of starting folic acid supplementation turned out to be statistically significant (*p* < 0.05). The reference period of supplementation was the period before pregnancy; it turned out that supplementation started at any other date is associated with the risk of heart defects in the child. Thus, the lack of supplementation at all is associated with an increase of this risk by more than four times compared to those taking supplements before pregnancy (OR = 4.19; *p* = 0.0117), whereas supplementation beyond the 8th week increases the risk almost threefold (OR = 2.90; *p* = 0, 0474). Additionally, supplementation from weeks 1–8 increases this risk, but not significantly (OR = 1.81; *p* > 0.05).

The total intake of folates combined with folic acid supplementation was assessed and compared with the recommendations/norm intake in group A (tested) and B (control). In the case of the group of women with fetal heart defects, mothers were significantly less likely to have folic acid intake and supplementation below the norm than women from the control group (22.8% vs. 10.7%). The calculated odds ratio was 2.25, which proves that the consumption/supplementation norms were met more than twice as rarely as in the control group. Folic acid intake above the norm was significantly more frequent in the control group (B) than in the study group (A), at 28.9% vs. 20.2%.

Additionally, folate intake enriched with folic acid supplementation before pregnancy was compared with the recommendations for women who did not use supplementation before pregnancy. Lack of supplementation before pregnancy and failure to meet the norm (insufficient folate intake within their diet, i.e., one below recommended levels) and the chi-square independence test also showed a statistically significant relationship (*p* < 0.05). Supplementation before pregnancy and meeting the norm was significantly less common in the study group than in the control group, at 12.7% vs. 24.0%. OR = 0.48, so it turned out that a diet providing folates + folate supplementation, allowing women to reach recommended levels, were twice as rare in group A than in group B. The detailed results are presented in Table 3.

The fact that pregnant women smoke tobacco is worth noting. In the entire group, 13.9% of the surveyed women declared smoking during pregnancy (despite the diagnosis of pregnancy; after 4 weeks, 6.8% of women still continued smoking). There was no statistically significant relationship between maternal cigarette smoking and the occurrence of heart defects in the fetus (*p* > 0.05). It is worth noting, however, that smoking during pregnancy, compared to people who did not smoke before pregnancy, was associated with the risk of defects in the child, although not significantly, OR = 1.80 (*p* > 0.05).

A total of 17.7% of the surveyed group declared alcohol consumption, of which as many as 12.8% of respondents continued to drink. Women drinking alcohol did not significantly affect the occurrence of a heart defect in the child (*p* > 0.05). Detailed data are included in Table 4.

There was a statistically significant difference in the trimester of pregnancy in which the women were examined (*p* < 0.001). It turned out that in group A, as many as 92.4% of mothers examined were in the third trimester, while in group B, there were 69.4% of such mothers.

The chi-square test of independence did not show a statistically significant relationship between maternal age as a risk factor and the occurrence of heart defects in the fetus (*p* > 0.05). However, taking the mother’s age below 25 years as a reference and calculating the odds ratios for individual age groups, it was found that fetal heart defects were almost four times more common in children of mothers aged 25–29 than in the youngest mothers (OR = 3.94; *p* = 0.0477). Additionally, heart defects in children in the group of mothers aged 30–34 and in those aged 35 or more turned out to be more common than in the youngest mothers. The corresponding odds ratios are OR = 3.20 and OR = 2.91. However, in the latter cases, no statistical significance was found.

There was no statistically significant relationship between the mother’s education level and the occurrence of CHD in children (*p* > 0.05). However, it is worth noting that in the case of mothers with vocational education, defects in their children occurred more than 2.5 times more often than in mothers with higher education (OR = 2.54).

Similarly, there was no statistically significant effect of father’s education on the occurrence of heart defects in the child (*p* > 0.05).

Additionally, the mother’s place of residence did not have a statistically significant impact on the occurrence of heart defects in the child (*p* > 0.05), although in the case of living in a small or medium-sized city, the risk of CHD in children was almost twice as frequent as in those living in a large city; the corresponding odds ratios are OR = 1.71:OR = 1.90). The detailed results are presented in Table 5.

Based on pre-pregnancy weight and height data, BMIs were calculated and compared to the standards proposed by the WHO [29]. Malnutrition before pregnancy was present in 3.8% of women from the study group [A] compared to 2.5% of women from the control group [B], normal body weight in relation to height occurred in 58.2% of women from group A compared to 52.1% of women from group B; however, excess body weight was diagnosed in approximately 38.0% of women in the study group and 45.5% of women in the control group.

There was no statistically significant relationship between the occurrence of heart defects in the fetus and the nutritional status before pregnancy, as measured by BMI (*p* > 0.05).

The occurrence of CHD in the fetus is statistically significantly dependent on the presence of heart defects or other in the family (*p* < 0.05). It turned out that if a congenital malformation was present in the mother, father or sibling, the risk of the defect occurring in the fetus was more than three times higher than in the case when there was no family history of the defect (OR = 3.27: *p* = 0.0294). Additionally, if the defect is present in a grandmother, grandfather, aunt, uncle and cousins, the risk of the defect in the child increases by more than 2.5 times compared to people who do not have a defect in the family (OR = 2.57; *p* = 0.0203).

There was no statistically significant impact of the presence of diabetes in the mother before pregnancy, gestational diabetes, hypertension before pregnancy, hypertension during pregnancy, hypothyroidism before pregnancy and other thyroid diseases on the occurrence of heart defects in the child (*p* > 0.05). The odds ratios did not differ significantly from unity.

Mothers of children from the control group took medications significantly more often than from the CHD group (*p* < 0.001). There was no statistically significant impact on the occurrence of heart defects in the child of medications containing iodine, heparin, Acard, insulin, or other medications (*p* > 0.05).

However, it was found that the mother’s use of Duphaston and Lutein reduced the risk of heart defects in the child by almost three times compared to people not taking any drugs; the corresponding odds ratios are (OR = 0.31; *p* = 0.0206) and OR = 0.33; *p* = 0.0397). The results of the analysis are presented in Table 6.

As shown in Table 7, a comparison of folate dose and time of supplementation of folic acid was carried out, and there were no significant differences in the relationship between the dose of folates and the time of their supplementation in our group. This particular relationship must be evaluated on a larger group of patients in further studies.

## 4. Discussion

A properly balanced diet determines the proper development of the fetus and the course of pregnancy and ensures the health of the mother. Nutritional recommendations clearly indicate the particular importance of increased demand for energy, protein, long-chain polyunsaturated fatty acids [LC-PUFA, especially *n*-3 acids], and many vitamins [folic acid, vitamins D, A, E, C, PP, B6] and minerals [iron, iodine, zinc, magnesium] [1,2,3,30,31,32].

The current recommendations and guidelines of most medical associations and WHO statements emphasize the importance of a balanced/proper diet, which should be implemented as a priority in ensuring the appropriate amount of energy and nutrients. Routine dietary supplementation to be taken by all pregnant women is not recommended, but additional supplementation should be provided when necessary [33].

What must be also emphasized is that balanced nutrition is important throughout pregnancy, but it should also be remembered that it becomes more important in the periconceptional period, because the period before pregnancy is crucial for the health of the woman and her child. Therefore, at this stage it is important to monitor the mother’s diet to ensure sufficient metabolism and proper development of the fetus through proper intake of energy and key nutrients [33]. It should also be remembered that intervention strategies among poorly nourished women are more effective if they are implemented several months before conception [3,33].

It is therefore very important that eating habits are strongly established already at the pre-pregnancy stage to ensure good nutrition from the first weeks of pregnancy [34].

Deficiencies during pregnancy are also related to the fact that the mother is in a poorer nutritional state at the beginning of pregnancy, so striving for a balanced prenatal diet and/or administering dietary supplementation are very valuable [33,34,35].

Every woman should prepare for pregnancy by striving to obtain the correct body weight, because underweight or excessive body weight in the periconceptional period and pregnancy carries the risk of many adverse health consequences for the fetus [36] and the mother [32]. Maternal obesity may cause neural tube and heart defects in the fetus or other complications [35,36,37,38]. Less than 50% of women change their eating habits during pregnancy [39], but any changes made are only minor [40].

Therefore, women who are planning a pregnancy, as well as pregnant women, should be the recipients of various activities in the field of health promotion as well as health and nutrition education in order to expand their knowledge about the impact of the mother’s nutritional status on the developing fetus and the saturation of the body with important nutrients [36]. It should also be noted that, unfortunately, a customary diet is not always able to cover the increased demand for essential ingredients during pregnancy, including vitamins and minerals [41,42]. In order to supplement the diet, the Polish Society of Gynecologists and Obstetricians (PTGiP) recommends supplementing five ingredients: folic acid, vitamin D, DHA, iron, and iodine [43].

### 4.1. Folic Acid—Dietary Intake and Supplementation vs. CHD

According to the recommendations of the PTGiP from 2020 [43] and the latest guidelines as of 2024 on folic acid supplementation [44], it is recommended that all women of reproductive age should use folic acid or combined supplementation (folic acid + active form of 5-methyltetrahydrofolate 5-MTHFR) for a period of at least 12 weeks before conception. The recommended dose is 0.4 mg/day for folic acid supplement or 400 μg of folic acid + 400 μg of 5-MTHF/day as a supplement to a natural folate-rich diet.

Folic acid deficiency in women, both before and during pregnancy, has negative health consequences. Women with a low supply of folate in the diet and lack of folic acid supplementation, especially at reproductive age, are at increased risk of developing a deficiency of this nutrient in the body [45]. During pregnancy, folic acid deficiency in women may increase the incidence of neural tube defects, heart defects, and other developmental disorders in newborns. The appropriate content of folic acid is necessary for the proper development of the fetal cardiovascular system, and this is what should be emphasized here in relation to our study [24,46,47,48].

A review of scientific research indicates that folic acid deficiency in the first 2 to 3 weeks of human pregnancy may lead to the development of heart defects in the fetus. This period can be considered a high risk of congenital heart defects and neural tube defects, because about 50% of pregnancies are unplanned, meaning the woman is usually not aware of her pregnancy and may not yet take preventive measures to protect the developing embryo [47,49].

Taking folates with the diet and appropriate folic acid supplementation at the beginning of pregnancy (or preferably before the planned pregnancy) reduces the incidence of developmental defects, including heart defects [16,33,50].

The best sources of folate in the diet are products of plant origin such as legumes (soybeans, beans, peas, broad beans), green leafy vegetables (spinach, parsley, asparagus, kale, broccoli, cabbage, lettuce), and whole grains and citrus fruits (oranges, mango, avocado). Products of animal origin such as ripening cheese, eggs, and some species of fish (e.g., tuna, salmon) contain a low content of folate; however, folates are found in larger amounts in the liver [1,50,51,52].

By reviewing the literature, we found that the average intake of folates in the diet by women of reproductive age or at the beginning of pregnancy is only approximately 50% of the recommended amount [53]. The recommended amount for pregnant women is 600 µg/day [1]. In this study, the average intake of folate by pregnant women from the study group [A] was estimated at 392.4 µg, and in the control group [B] was 428.1 µg, values that are 65.4% vs. 71.4%, respectively, of the recommended amount. Many Polish [52,54,55] and foreign [56,57,58] studies assessing/monitoring the diet of pregnant women or women planning to become pregnant show insufficient intake of dietary folates.

The use of supplementation by women planning a pregnancy or pregnant women is implemented to a limited extent. Dietary folic acid supplementation in our study was applied only in 38.4% of women before pregnancy, including only 13.2% from group A and 25.2% of women from group B. In a study by Grzelak et al. [59], folic acid was taken by 43% of respondents who were pregnant women or planning pregnancy. A similar situation occurred in the study by Wierzejska and Wojda [60], where folic acid was taken by only 42% of the surveyed women before becoming pregnant, while during pregnancy, supplementation increased to 83%. However, most women started supplementation only from the 5th–6th week of pregnancy. In other Polish studies [61], which included students from the Podkarpackie Voivodeship, it was found that 52.4% of young women did not supplement folic acid before planning a pregnancy.

Folic acid supplementation before conception is different all over the world. According to Toivonen et al. [62], in the period before pregnancy, folic acid supplementation was used as follows: 32–51% in North America, 9–78% in Europe, 21–46% in Asia, 4–34% in the Middle East, 32–39% in Australia/New Zealand, and 0% in Africa.

Dietary supplementation makes up for some nutritional deficiencies. Pregnant women living in the Netherlands, according to Borg et al. [63], seem to have adequate intake of essential nutrients; however, in the case of folic acid and vitamin D, supplementation was necessary to achieve the recommended intake. However, the vast majority of Polish women in the study by Jankowska et al. [55] did not meet targets (EARs) for the most essential elements and vitamins with their diet. Insufficient intake of folic acid was noted for over 80% of women, but significant improvements were achieved with folic acid supplementation (93% of the population met the EAR of total intake with supplementation).

A pregnant woman’s diet is crucial for the growth and development of the fetus. The results of the study conducted by Yang et al. [64,65] showed the protective role of a more diversified and nutrient-rich diet on the reduced risk of fetuses with complete CHD. It has been shown that a higher-quality diet rich in vitamins [folic acid, niacin] and minerals [iron, selenium] may have a protective effect against the development of congenital heart defects in the fetus. Moreover, according to the authors [66], pregnant women with a better diet quality may have greater opportunities to acquire nutritional knowledge and pay more attention to healthy eating and may thus have a better nutritional status.

In other studies, Dolk et al. [15] analyzed individual dietary components and consumption of oranges and other fruits rich in folic acid at least three times a week and found a significantly lower risk of CHD among the studied cases. The vast majority of women in this study took folic acid supplements at the end of the first trimester. The authors found no risk of CHD in the small group of women who did not take supplements at all, or among women who started taking supplements after 6 weeks of pregnancy. These results confirm the role of maternal diet in the etiology of CHD. An incorrect diet low in fruits and vegetables with a daily intake of carbonated and high-energy drinks or other foods was associated with a 56% excess risk of CHD in the study population. Researchers also point out the need for further research on diet.

In our own study, a joint assessment of dietary folate intake and the use of folic acid supplementation among the surveyed women was made. It was shown that the use of a folic acid supplement in both the study and control groups significantly improved the recommended intake of this ingredient among pregnant or pre-pregnant women. However, it was noticed that low dietary folate intake in the absence of supplementation, especially in the periconceptional period, did not meet the daily intake target and was significantly less frequent in the study group than in the control group.

In our study, the time of introduction of folic acid supplementation by the study population turned out to be statistically significant. Women who supplemented folic acid before conception compared to those who did not use supplementation or started supplementation later [after 8 weeks of pregnancy] had a significantly higher risk of developing fetal heart defects. The period between the third and eighth week of pregnancy is a critical period of heart formation in the fetus [67,68].

In the study by Mao et al. [24], supplementation and dietary intake of folic acid were assessed simultaneously in relation to the risk of CHD. They found that women who did not take a folic acid supplement and had lower dietary folate intake had an almost 2-fold increased risk of CHD in their children. A synergistic effect of dietary folate intake and folic acid supplementation was also observed. It was shown that a longer duration of supplementation had a beneficial effect on reducing the overall risk of CHD.

Similar results to those in our own work were obtained in the study by Liu et al. [16]. In the control group, a higher percentage of pregnant women supplementing folic acid was found compared to the study group (93.1% vs. 84.8%), although the percentages of women with supplementation were much higher than in our study. Moreover, the lack of maternal folic acid supplementation was associated with an increased risk of total CHD and ASD. It was proven that in the entire CHD group, folic acid supplementation in the first and second trimester was associated with a significantly higher risk of congenital heart defects in the children compared to women’s supplementation during the three months before conception. At the same time, according to the authors, folic acid supplementation may reduce the risk related to genetic mutations.

Properly selected folic acid supplementation started before conception may have strong antithrombotic and antioxidant properties and lead to improvement of endothelial dysfunction or prevention of low folic acid levels in the mother, which may lead to the accumulation of homocysteine and interfere with the proper development of the cardiac neural crest [16,24].

Folic acid supplementation in the periconceptional period in women reduced the risk of congenital heart defects and its subtypes in children, which was also observed in other studies [14,69,70,71], although in some studies, no significant relationship was found [72]. However, use of supplementation was shown to have a protective effect [15,73,74].

It is also worth mentioning the results of the review and meta-analysis conducted by Cheng et al. [75]. The authors emphasized that the data from the analysis suggest that folic acid supplementation in the mother is associated with a reduced risk of heart defects in the fetus; however, the heterogeneity of this relationship is high. The differences result mainly from the methods used, geographical differences [Europe, China, America], and time and dose of folic acid use. The authors suggest that further research is needed on the reasonable timing and dosage of folic acid.

According to Ledowsky et al. [76], women of reproductive age do not obtain sufficient intake of folic acid from food sources alone, even if they consume enhanced food products. However, by also taking folic acid supplements, they may exceed the upper tolerable limit of folic acid intake to 1000 mcg. Therefore, dietary supplementation should be carefully planned to compensate for inadequate nutrient intake [including folic acid] and also to avoid exceeding the upper tolerable limit of the UL.

It has long been known that maternal dietary patterns and nutritional status influence both maternal and child health outcomes. It has been concluded that a pregnant woman’s adherence to a higher-quality diet reduces oxidative damage to the mother’s DNA and lipid oxidation, which may additionally have a beneficial effect on the proper development of the fetal cardiovascular system. Improper diets in pregnant women may also be a mediator of metabolic diseases, including gestational diabetes and hypertension during pregnancy, which additionally affect the cardiovascular system of the fetus [64,77].

All women planning conception and pregnancy should be encouraged to eat a balanced diet with the best possible and varied composition in accordance with recommendations. Regular consumption of fresh fruit, vegetables, dairy products, fish, and seafood with a low mercury content should be encouraged because, based on available data, mothers with such dietary patterns are less susceptible to the occurrence of congenital heart defects in general and some subtypes [15,65,78]. It has also been proven that mothers with excessive consumption of fried, smoked, grilled, and pickled vegetables [66] and frequent daily consumption of carbonated and high-energy drinks [15] are at greater risk of fetal CHD and ventricular septal defects (VSD).

### 4.2. Other Risk Factors for CHD

Research shows that many women lead unhealthy lifestyles when they enter pregnancy, which are characterized by poor diet quality, low levels of physical activity, smoking, and excessive alcohol consumption, and which remain common at the time of conception. Unfortunately, most women do little to change their lifestyle [50,79,80,81,82].

Every woman planning a pregnancy should be aware of the negative impact of harmful substances (including alcohol and nicotine) on her body and the baby. These substances cause serious damage to the central nervous system, weakening of the immune system, heart defects, premature birth, and low birth weight of the newborn [15,74,83,84].

However, what is worrying is the fact that researchers [15,39,63,83] have reported abnormal behavior among women regarding alcohol consumption during pregnancy (from 17% up to 54%). Similarly, it is also stated that despite becoming pregnant, approximately 4.4–19.8% of women declare smoking [15,16,39,74,83]. In our study, we also found abnormal habits. As many as 17.7% of women consumed alcohol and almost 14% smoked while pregnant.

We found no significant relationship between alcohol consumption and smoking and an increased risk of heart defects in the fetus among the surveyed women. This may have been due to the study group being too small compared to the control group. It is worth noting, however, that smoking during pregnancy, compared with women who did not smoke before pregnancy, increased the risk of defects in the child, although not significantly. Studies have shown a relationship with alcohol consumption [16,74] or smoking [16,74,85,86] and the occurrence of heart defects in the children, but some studies have also shown no such relationship [15,87,88].

Currently, the incidence of diabetes among women of reproductive age is increasing worldwide. The diabetic intrauterine environment can cause placental dysfunction and hormonal changes, leading to congenital defects, and has long been known as a risk factor for congenital heart disease [89,90]. Gestational diabetes is one of the most common pregnancy-related endocrinopathies, affecting 9% to 31.5% of pregnancies worldwide [77,82], closely following the prevalence of diabetes and obesity in the general population [91] due to changes in lifestyle and diet structure [77]. Studies conclude that gestational diabetes is associated with a lower risk of CHD than pre-pregnancy diabetes [87] because hyperglycemia has a more critical impact on heart development in early pregnancy than in a later period of pregnancy [89]. The research of Depla et al. [91] confirms the relationship between the occurrence of heart defects in the fetus and diabetes occurring in the mother before pregnancy, but also with gestational diabetes, as found in the study by Liu et al. [16]. In the study by Kawai et al. [87], as in our study, no such relationship was found.

Thyroid disorders are quite common in women of reproductive age. Hypothyroidism occurs in 4% of pregnancies and hyperthyroidism in 2.4%. Thyroid hormones are necessary to maintain normal pregnancy and fetal development [92,93]. Important in the context of fetal heart defects is the risk of teratogenicity, which should be taken into account when choosing antithyroid drugs during pregnancy and the preconception period. Exposure to methimazole or carbimazole is associated with more serious birth defects, including ventricular septal defects, while exposure to propylthiouracil is associated with less severe defects. Therefore, it is important to avoid overtreatment of hyperthyroidism in pregnancy [92].

One of the diseases during pregnancy that has been assessed as a risk factor for CHD is maternal arterial hypertension, which occurs in approximately 2–10% of pregnant women [94]. The mechanisms by which hypertension or antihypertensive medications may increase the risk of CHD have not been fully defined. It has been proposed that both maternal hypertension and antihypertensive medications may cause uteroplacental insufficiency by reducing blood flow to the uterus during pregnancy, also lowering fetal blood pressure [94]. The authors suggest that the risk of CHD in the fetus was approximately 80% higher among women with hypertension compared to women without hypertension [94], which was not confirmed in our research.

### 4.3. Strengths and Limitations Should be Taken into Account When Interpreting Study Results

This study has several strengths. The study collected detailed data on the supplementation of folic acid [before pregnancy, at the beginning and after the eighth week of pregnancy] as well as the consumption/analysis of these ingredients in the diet, which allowed these variables to be controlled for each other. Cumulative intake and supplementation in the context of CHD were also studied. The mother’s eating habits are usually stable throughout pregnancy. The mother’s diet throughout pregnancy is comparable to the diet in the third–eighth week of pregnancy, which is a critical period of fetal cardiovascular development [68]. Another strength of the study was the collection of clinical and nutritional history and accurate ultrasound diagnosis in the study and control groups at the same time, which allows for direct comparison. Another strength of the study was the inclusion of many risk factors [socio-demographic factors, nutritional status, occurrence of diseases, drug use, alcohol consumption, and smoking] and the occurrence of genetic diseases in the family and taking them into account as confounding factors.

The weaknesses of the study include its limited sample size (mainly that of the study group), which meant it was not possible to separately examine the associations of dietary intake and supplementation with other subtypes of CHD, which may have etiological heterogeneity [64]. We also could not exclude the possibility of underestimation or consumption of larger portions of food by the patients (despite the help of the interviewer and available tools illustrating the size of the portions) or the interference of unknown factors. We also took into account the possibility of recall/memory bias. FFQs may be subject to inter-rater errors, yet this questionnaire remains the most frequently used tool in survey research [95]. Case–control studies are among the most reliable studies analyzing the relationship between disease risk factors, but they may be susceptible to memory errors. Pregnant women may be biased or incorrectly select answers [74].

Our study can help identify risk and protective factors of congenital heart defects in the fetus in women planning a pregnancy and/or at the beginning of pregnancy with a low intake of folic acid or other nutrients [vitamin D, omega-3 fatty acids] to encourage a more balanced diet consistent with recommendations and, in some cases, enhanced with supplementation. This will help develop appropriate preventive programs aimed at improving the intake of these nutrients in women of reproductive age. However, further research on a larger study and control group is needed.

## 5. Conclusions

A history of congenital heart defects, lack of folic acid supplementation before pregnancy, and lack of dietary supplementation before pregnancy were associated with congenital heart defects in the fetus. Place of residence, parents’ education, lifestyle habits such as smoking, alcohol consumption, nutritional status before pregnancy, and mother’s diseases did not show a significant relationship with congenital heart defects in the children. There is an urgent need to develop preventive strategies and conduct extensive public education.

## Figures and Tables

**Table 1:** Average age of pregnant women and average age of fetuses in the study.

Variables	CHD (A)		NHA (B)		Test Value, *p*
	$\bar{x}$	SD	$\bar{x}$	SD	
**Age of women (years)**	31.9	5.3	31.4	5.6	*Z* = 0.384; *p* = 0.701
Age of fetuses (week of gestation)	29.6	5.6	26.7	5.4	***Z* = 3.539; *p* = 0.0004**

Qualitative variables were analyzed in both study groups.

**Table 2:** Comparison of the mean values of selected variables in the group of women with heart defects in CHD (congenital heart defect) fetuses (A) and in the group of women without heart defects in NHA (normal heart anatomy) fetuses (B).

Variables	Group								Test Value *Z*	Significance *p*
	CHD (A) (*n* = 79)				NHA (B) (*n* = 121)					
	N	$\bar{x}$	Me	SD	N	$\bar{x}$	Me	SD		
**BMI before pregnancy**	**79**	24.7	23.4	5.2	121	25.9	24.2	6.3	1.255	0.21
Folate diet [µg]	79	392.4	372.2	129.2	121	428.1	409.4	149.0	1.739	0.0820
Folic acid supplementation [µg]	64	463.3	400.0	178.0	112	492.4	400.0	193.7	0.735	0.462
Folate diet + supplementation [µg]	79	543.6	472.5	365.2	121	614.5	589.2	374.0	1.442	0.149

**Table 3:** A relation between maternal risk factors (fulfillment of the objectives related to folates in diet, folic acid supplementation and total intake) and congenital heart defects in fetuses compared with the control group—univariate logistic regression analysis. CHD = congenital heart defect.

Factors/Variables	CHD Cases A (*n* = 79)		Controls B (*n* = 121)		
	No.	%	No.	%	OR (95%CL)
Folates—dietary intake					
Implementation below the norm	65	82.28	88	72.73	1.48 (0.69–3.19)
Implementation normal	12	15.19	24	19.83	Ref.
Implementation above the norm	2	2.53	9	7.44	0.44 (0.08–2.41)
	chi^2^ = 3.235; *p* = 0.198				
Folates = diet + supplements [total]					
Implementation below the norm	18	22.8	13	10.7	2.25 (1.02–5.03)
Implementation normal	45	57.0	73	60.2	Ref.
Implementation above the norm	16	20.2	35	28.9	0.74 (0.37–1.49)
**chi^2^ = 5.972; *p* = 0.050**					
Folates—implementation of the pre-pregnancy norm = diet + supplementation					
Supplementation before pregnancy and implementation the norm	10	12.7	29	24,0	Ref.
Lack of supplementation before pregnancy and not meeting the norm	69	87.3	92	76,0	0.49 (0.22–1.07)
**chi^2^ = 3.894; *p* = 0.0485**					
Folic acid supplementation					
Supplementation before pregnancy	10	13.16	29	25.22	Ref.
Supplementation from 1–8 weeks of pregnancy	40	52.63	64	55.65	1.81 (0.79–4.14)
Supplementation above the 8th week of pregnancy	13	17.11	13	11.30	**2.90 (1.01–8.36)**
No supplementation	13	17.11	9	7.83	**4.19 (1.37–12.84)**
	**chi^2^ = 7.831; *p* = 0.0496**				

Basic data on selected lifestyle elements were obtained from the medical records and the survey and were analyzed.

**Table 4:** The relationship between maternal risk factors (selected lifestyle elements) and congenital heart defects in fetuses compared to the control group, assessed by univariate logistic regression analysis. CHD = congenital heart defect.

Factors/Variables	CHD Cases A (*n* = 79)		Controls B (*n* = 121)		
	No.	%	No.	%	OR (95%CL)
Cigarettes					
Did not smoke before pregnancy	64	81.01	101	83.47	Ref.
Smoked until the 4th week of pregnancy	6	7.59	12	9.92	0.80 (0.28–2.26)
Smokes in pregnancy	9	11.39	8	6.61	1.80 (0.66–4.95)
	chi^2^ = 1.607; *p* = 0.448				
Alcohol					
Did not drink before pregnancy	66	83.54	90	74.38	Ref.
Consumed alcohol until the 4th week of pregnancy	5	6.33	7	5.79	0.97 (0.29–3.22)
Currently drinking	8	10.13	24	19.83	0.45 (0.19–1.08)
	chi^2^ = 3.354; *p* = 0.187				

**Table 5:** The relationship between selected maternal risk factors (age, mother’s and father’s education, place of residence) and congenital heart defects in fetuses compared to the control group, assessed by univariate logistic regression analysis. CHD = congenital heart defect.

Factors/Variables	CHD Cases A (*n* = 79)		Controls B (*n* = 121)		
	No.	%	No.	%	OR (95%CL)
Trimester of pregnancy					
2	6	7.59	37	30.58	Ref.
3	73	92.41	84	69.42	**5.36 (2.13–13.50)**
	**chi^2^ = 14.959; *p* = 0.0001**				
Mother’s age					
<25	3	3.80	14	11.57	Ref.
25–29	27	34.18	32	26.45	3.94 (1.01–15.3)
30–34	24	30.38	35	28.93	3.20 (0.82–12.5)
≥35	25	31.65	40	33.06	2.91 (0.75–11.3)
	chi^2^ = 4.429; *p* = 0.219				
Mother’s education	**177**				
Basic	2	3.08	6	5.36	0.68 (0.13–3.58)
Vocational	5	7.69	4	3.57	2.54 (0.63–10.2)
Medium	25	38.46	35	31.25	1.45 (0.75–2.82)
Higher	33	50.77	67	59.82	Ref.
	chi^2^ = 3.074; *p* = 0.380				
Father’s education	152				
Basic	1	1.75	4	4.35	0.33 (0.03–3.18)
Vocational	12	21.05	13	14.13	1.22 (0.48–3.1)
Medium	19	33.33	38	41.30	0.66 (0.31–1.39)
Higher	25	43.86	37	40.22	Ref.
	chi^2^ = 2.407; *p* = 0.492				
Place of residence					
Village	20	25.64	35	29.17	1.08 (0.53–2.21)
A small town	9	11.54	10	8.33	1.71 (0.62–4.7)
Average city	19	24.36	19	15.83	1.90 (0.87–4.14)
Big city	30	38.46	56	46.67	Ref.
	chi^2^ = 3.241; *p* = 0.356				

The analyzed factors included the occurrence of defects in the family [genetic factors] and the mother’s nutritional status before pregnancy [malnutrition, normal, excess body weight], diseases in mothers [diabetes, hypertension, hypothyroidism, or other thyroid diseases], and medications taken.

**Table 6:** Association between maternal risk factors (nutritional status before pregnancy, occurrence of defects in the family, mother’s disease, medications taken) and congenital heart defects in the fetus compared to control group, as assessed by univariate logistic regression analysis. CHD = congenital heart defect.

Factors/Variables	CHD Cases A (*n* = 79)		Controls B (*n* = 121)		
	No.	%	No.	%	OR (95%CL)
Nutritional status before pregnancy					
Malnutrition	3	3.8	3	2.48	0.64 (0.15–2.73)
Standard	46	58.2	63	52.07	Ref.
Overweight	18	22.8	32	26.45	0.70 (0.35–1.40)
Obesity	12	15.2	23	19.01	0.67 (0.3–1.49)
	chi^2^ = 1.264; *p* = 0.738				
Occurrence of defects in the family					
Mother, father, siblings	10	13.70	6	5.45	**3.27 (1.12–9.55)**
Grandmother, grandfather	0	0	1	0.91	**2.57 (1.15–5.71)**
Aunt, uncle, cousins	17	23.29	12	10.91	
No defects	46	63.01	91	82.73	Ref.
	**chi^2^ = 10.595; *p* = 0.014**				
Occurrence of diseases in the mother					
Diabetes before pregnancy	3	4.23	4	3.60	1.18 (0.25–5.49)
Gestational diabetes	9	12.68	13	11.71	0.78 (0.14–4.43)
Insulin resistance	0	0.00	2	1.80	
No diseases	59	83.1	92	82.88	Ref.
	chi^2^ = 1.356; *p* = 0.716				
Hypertension before pregnancy	3	4.23	5	4.55	0.92 (0.21–4.04)
Hypertension during pregnancy	0	0	1	0.91	
No diseases	68	95.77	104	94.55	Ref.
	chi^2^ = 0.662; *p* = 0.718				
Hypothyroidism before pregnancy	13	18.31	29	26.13	0.63 (0.29–1.4)
Other thyroid diseases	3	4.23	5	4.50	1.08 (0.23–5.06)
No diseases	55	77.46	77	69.37	Ref.
	chi^2^ = 1.545; *p* = 0.462				
**Medicines**					
Medicines with iodine	15	21.74	29	27.62	0.80 (0.37–1.71)
	chi^2^ = 0.690; *p* = 0.406				
Duphaston	6	8.7	29	27.62	**0.31 (0.12–0.84)**
	**chi^2^ = 8.873; *p* = 0.0029**				
Lutein	5	7.25	24	22.86	**0.33 (0.1–0.95)**
	**chi^2^ = 7.032; *p* = 0.0080**				
Heparin and other medications	6	8.7	10	9.52	1.90 (0.52–7.63)
	chi^2^ = 0.029; *p* = 0.865				
Acard	9	13.04	19	18.1	0.75 (0.27–2.11)
	chi^2^ = 0.737; *p* = 0.391				
Insulin	3	4.35	5	4.76	1.25 (0.24–6.41)
	chi^2^ = 1.414; *p* = 0.235				
Other medications	8	11.59	21	20.0	0.61 (0.23–1.62)
	chi^2^ = 0.014; *p* = 0.906				
No medications	42	60.87	28	26.67	Ref.
	**chi^2^ = 18.938; *p* = 0.0000**				

**Table 7:** The relationship between foliate dose and foliate supplementation in a group of fetuses with congenital heart defects and control group. CHD = congenital heart defect.

Supplementation Time and Dose Relationship	Statistical Analysis
Supplementation **before** pregnancy compared with a median value of folate dose in CHD group and control group	*p* = 0.8786
Supplementation **between 1 and 8 weeks** of pregnancy compared with a median value of folate dose in CHD group and control group	*p* = 0.2124
Supplementation **after the 8th week** of pregnancy compared with a median value of folate dose in CHD group and control group	*p* = 0.3937

## Data Availability

The data presented in this paper are available upon request from the corresponding author. The data are not publicly available due to privacy and ethical restrictions.
